# Establishment of predictive nomogram and web-based survival risk calculator for desmoplastic small round cell tumor: A propensity score-adjusted, population-based study

**DOI:** 10.17305/bb.2022.8633

**Published:** 2023-05-01

**Authors:** Sihao Chen, Yu Pu, Yuzhu Jiang, Yingda Liu, Mengxia Li, Mingfang Xu

**Affiliations:** 1 Cancer Center, Daping Hospital, Army Medical University (Third Military Medical University), Chongqing, China

**Keywords:** Desmoplastic small round cell tumor (DSRCT), nomogram, survival, triple therapy, Surveillance, Epidemiology, and End Results (SEER)

## Abstract

Desmoplastic small round cell tumor (DSRCT) is a rare undifferentiated malignant soft tissue tumor with a poor prognosis and a lack of consensus on treatment. This study’s objective was to build a nomogram based on clinicopathologic factors and an online survival risk calculator to predict patient prognosis and support therapeutic decision-making. A retrospective cohort analysis of the Surveillance, Epidemiology, and End Results (SEER) database was performed for patients diagnosed with DSRCT between 2000 and 2019. The least absolute shrinkage and selection operator (LASSO) Cox regression analysis was applied to identify the individual variables related to overall survival (OS) and cancer-specific survival (CSS), as well as to construct online survival risk calculators and nomogram survival models. The nomogram was employed to categorize patients into different risk groups, and the Kaplan–Meier method was utilized to determine the survival rate of each risk category. Propensity score matching (PSM) was used to assess survival with different therapeutic approaches. A total of 374 patients were included, and the median OS and CSS were 25 (interquartile range 21.9–28.1) months and 27 (interquartile range 23.6–30.3) months, respectively. The nomogram models demonstrated high predictive accuracy. PSM found that patients with triple-therapy had better CSS and OS than those who received surgery plus chemotherapy (median survival times: 49 vs 34 months and 49 vs 35 months, respectively). The nomogram successfully predicted the DSRCT patients survival rate. This approach could assist doctors in evaluating prognoses, identifying high-risk populations, and implementing personalized therapy.

## Introduction

Desmoplastic small round cell tumor (DSRCT) is a rare undifferentiated soft tissue malignant tumor associated with EWS-WT1 fusion protein produced by t (11;22) (p13;q12) chromosomal translocation [1–4]. First described by Gerald and Rosey in 1989, this tumor has an insidious onset and primarily arises in adolescent and young adult males [5, 6]. DSRCT has no specific clinical manifestations, and the common symptoms are mainly abdominal and pelvic mass and pain, making it difficult to diagnose, and most patients have already metastasized at the time of diagnosis [3, 4]. The 5-year overall survival (OS) rate is a dismal 15%–25%, and the median survival time is only about 2 years [7, 8].

Due to its small round cell tumor characteristics, DSRCT was often misdiagnosed as Ewing sarcoma (ES) until it was clear that it was a distinct class of malignancy [9]. Up to now, the treatment methods of DSRCT mainly include surgery, chemotherapy, and radiotherapy, and the chemotherapy regimen mainly refers to ES [10, 11]. Chemotherapy is the preferred treatment for most DSRCT patients due to its sensitivity to initial chemotherapy. The commonly used chemotherapy regimen for DSRCT patients, P6 chemotherapy regimen, consists of alternating vincristine/doxorubicin/cyclophosphamide and etoposide/ifosfamide (VDC/IE) [12, 13]. Unfortunately, the OS of DSRCT patients was substantially lower than that of ES patients, suggesting that DSRCT and ES have different biological backgrounds [14, 15]. Surgical treatment is the only potentially curative therapy for DSRCT; however, the R0 resection is difficult to achieve due to multiple serosal tumor nodules and obscure boundaries [16]. Postoperative quality of life is also an issue, as surgical resection is often extensive, involving the primary tumor, peritoneum, lymph nodes, and adjacent tissue [17]. Studies have shown that patients who receive whole abdominal radiotherapy after surgery have better OS, which can further improve the local control rate [18]. However, radiotherapy is not widely used in DSRCT, and its therapeutic contribution must be further clarified.

**Figure 1.**
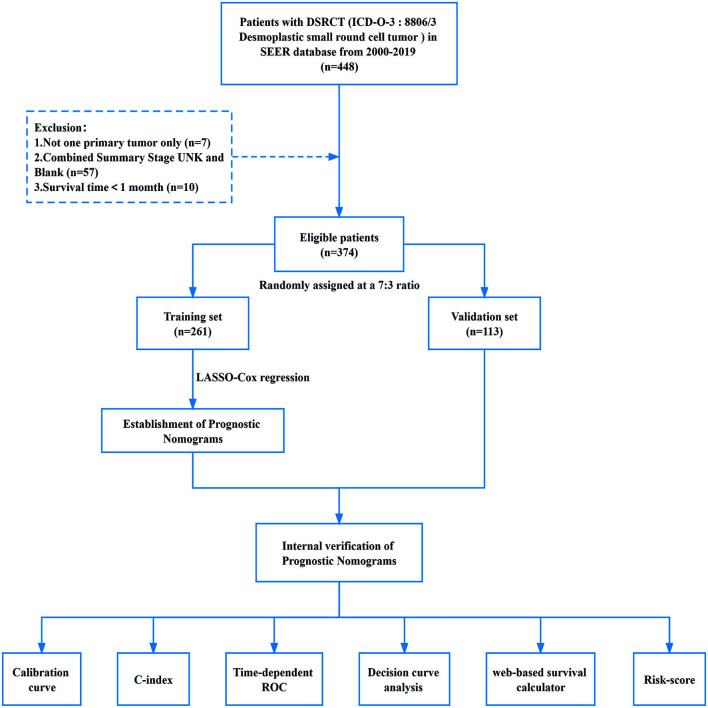
**Study design and the workflow diagram.** DSRCT: Desmoplastic small round cell tumor; ROC: Receiver operating characteristic; C-index: Concordance index; SEER: Surveillance, Epidemiology, and End Results; LASSO: Least absolute shrinkage and selection operator.

Accurate staging of tumors is important for treatment decision-making. Unfortunately, there is currently no recognized DSRCT staging. The American Joint Committee on Cancer (AJCC) sarcoma staging and peritoneal carcinoma index (PCI) are two commonly used clinical staging methods [19, 20], but their accuracy needs to be further verified. We designed and validated a potent survival risk rating system based on large-scale population data and available clinicopathological factors to enhance clinical decision-making and improve the existing prognostic systems.

## Materials and methods

### Study design and selection criteria

The selection criteria included: (1) DSRCT patients diagnosed between 2000 and 2019, histopathological coded as 8806/3 with the primary tumor only, per the International Classification of Diseases for Oncology, Third Edition (ICD-O-3); (2) Patients with a survival time > 1 month; (3) Patients with complete follow-up data; (4) Patients with information on survival months, vital status, sex, age, race-site of laterality, marital status, median household income - 2019 inflation adjusted, combined summary stage, collaborative stage (CS) tumor size and primary tumor therapy regime (Figure 1). Eligible patients were arbitrarily separated into validation and training groups (3:7 ratio). The endpoints of this study were cancer-specific survival (CSS) and OS. DSRCT CSS and OS were calculated using the Surveillance, Epidemiology, and End Results (SEER) variables of cause-specific death classification and other cause-of-death classifications. Various treatment-related areas were searched for information, including radiation recode, chemotherapy recode, and the reasons for no cancer-directed surgery.

### Ethical statement

The research was conducted per the Declaration of Helsinki and the transparent reporting of a multivariable prediction model for individual prognosis or diagnosis (TRIPOD) reporting standards for prognostic studies. The need for informed consent was eliminated since the SEER database is freely accessible online, and all data has been anonymized and de-identified. The SEER* Stat tool V8.4.0 was employed to collect patient information from the most recent SEER database (https://seer.cancer.gov).

### Statistical analysis

The population data collected from the SEER database were arbitrarily divided into 3:7 validation and training groups. We used the chi-square test to analyze the frequencies and percentages reported for all categorical variables. The log-rank test was used to compare survival curves constructed using the Kaplan–Meier method. A penalized Cox proportional hazard model utilizing the least absolute shrinkage and selection operator (LASSO) was employed in the development cohort to detect OS and CSS-related covariates. After identifying and evaluating independent prognostic indicators (*P* < 0.05), a prognostic nomogram was built and utilized to illustrate the risk model in an online survival calculator.

The area under the time-dependent receiver operating characteristic (ROC) curve and concordance index (C-index) was used to assess discrimination. To assess the congruence between the expected and actual survival probabilities, calibration curves were developed. Using decision curve analysis and time-dependent ROC, we evaluated the predictive performance of our model with the combined summary stage (SEER-stage). Individual risk scores were determined using the predetermined nomograms. To further divide patients into low- and high-risk categories, the Surv-Cutoff function was used to determine the optimal thresholds for CSS and OS. Using heatmaps of risk factor relationships, the clinical features distribution in distinct risk categories for CSS and OS was shown. The variations in survival across the various treatments were examined using propensity score matching (PSM). All analyses were conducted using SPSS 26.0 and R software V4.1.1 (https://www.r-project.org/), along with the glment, rms, timeROC, survminer, ggplot2, ggRisk, ggDCA, Matching, shine, and DynNom packages. The significance level used was *P* < 0.05.

## Results

### Clinical characteristics of patients

From 2000 to 2019, there were 374 patients in the whole cohort of the SEER database who were diagnosed with DSRCT and met the inclusion criteria. After arbitrarily dividing them at 3:7 ratio, with 113 and 261 in the validation and training groups, respectively, their clinical characteristics were compared. The distribution of patients did not differ statistically significantly between the validation and training groups (*P* > 0.05). The patients’ clinical profiles and demographic attributes are shown in Table 1. The bulk of the patient population consisted of young patients (age ˂ 30: *n* ═ 267, 71.4%), male (*n* ═ 290, 77.5%), white (*n* ═ 245, 65.6%), of low household income (*n* ═ 257, 68.7%), and unmarried (*n* ═ 277, 74.1%). According to an examination of primary site codes in the SEER database, approximately 71.7% of patients had primary sites in the abdomen and pelvis. The highest percentage of patients having distant metastases, according to the SEER-stage system, was 69.3% (*n* ═ 259). Depending on the treatment, 52.1% of patients had surgery, 86.6% received chemotherapy, and 24.9% received radiation. In the SEER database, the median OS and CSS times were 25 (interquartile range [IQR] 21.9–28.1) months and 27 (IQR 23.6–30.3) months, respectively. In addition, the median OS and CSS were 25 (IQR 21.1–28.8) and 28 (IQR 23.5–32.5) months, respectively, in the training group, whereas they were 24 (IQR 18.4–29.6) and 26 (IQR 23.5–30.5) months, respectively, in the validation group.

**Table 1:** Characteristics of patients with desmoplastic small round cell tumor in the training and validation group

**Characteristics**	**Total (*n* ═ 374) *n* (%)**	**Training group (*n* ═ 261) *n* (%)**	**Validation group (*n* ═ 113) *n* (%)**	***P* value**
**Years of diagnosis**				0.394
2000–2009	167 (44.7)	112 (42.9)	55 (48.7)	
2010–2019	207 (55.3)	149 (57.1)	58 (51.3)	
**Age, years**				0.767
<18	126 (33.7)	90 (34.5)	36 (31.9)	
18–30	141 (37.7)	95 (36.4)	46 (40.7)	
>30	107 (28.6)	76 (29.1)	31 (27.4)	
**Sex**				0.865
Male	290 (77.5)	202 (77.4)	88 (77.9)	
Female	84 (22.5)	59 (22.6)	25 (22.1)	
**Race**				0.241
White	245 (65.5)	167 (64.0)	78 (69.0)	
Black	87 (23.3)	59 (22.6)	28 (24.8)	
Others	42 (11.2)	35 (13.4)	7 (6.2)	
**Household income, $**				0.291
<70000	257 (68.7)	185 (70.9)	72 (63.7)	
≥70000	117 (31.3)	76 (29.1)	41 (36.3)	
**Marital status**				0.514
Married	97 (25.9)	71 (27.2)	26 (23.0)	
Others	277 (74.1)	191 (73.2)	87 (77.0)	
**Grade**				0.315
Unknown	289 (77.3)	197 (75.5)	92 (81.4)	
III-IV	85 (22.7)	64 (24.5)	21 (18.6)	
**Primary site**				0.875
Abdomen/pelvis	268 (71.7)	187 (71.6)	81 (71.7)	
Others	106 (28.3)	74 (28.4)	32 (28.3)	
**Laterality**				0.699
Others	316 (84.5)	222 (85.1)	94 (83.2)	
Paired	58 (15.5)	39 (14.9)	19 (16.8)	
**Tumor size**				0.777
Others*	182 (48.7)	126 (48.3)	56 (49.6)	
>4 cm	192 (51.3)	135 (51.7)	57 (50.4)	
**SEER-stage**				0.862
Localized	45 (12.0)	32 (12.3)	13 (11.5)	
Regional	70 (18.7)	46 (17.6)	24 (21.2)	
Distant	259 (69.3)	183 (70.1)	76 (67.3)	
**Surgery**				0.478
Yes	195 (52.1)	132 (50.6)	63 (55.8)	
No/Unknown	179 (47.9)	129 (49.4)	50 (44.2)	
**Chemotherapy**				0.836
Yes	324 (86.6)	227 (87.0)	97 (85.8)	
No/Unknown	50 (13.4)	34 (13.0)	16 (14.2)	
**Radiation**				0.417
Yes	93 (24.9)	61 (23.4)	32 (28.3)	
No/Unknown	281 (75.1)	200 (76.6)	81 (71.7)	

*Others include ≤ 4 cm and unknown.

### Independent prognostic factors selection and nomogram construction

**Figure 2.**
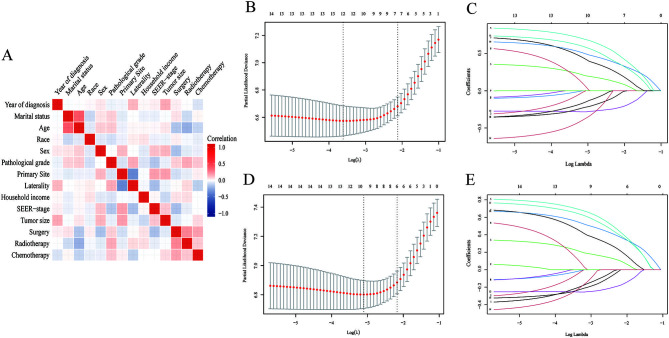
**Correlation analysis and LASSO regression model.** The results of correlation analysis between all included variables (A). Selection of tuning parameter (λ) for the LASSO model in OS (B) and CSS (D); LASSO coefficients of 14 features in OS (C) and CSS (E). OS: Overall survival; CSS: Cancer-specific survival; LASSO: Least absolute shrinkage and selection operator.

**Figure 3.**
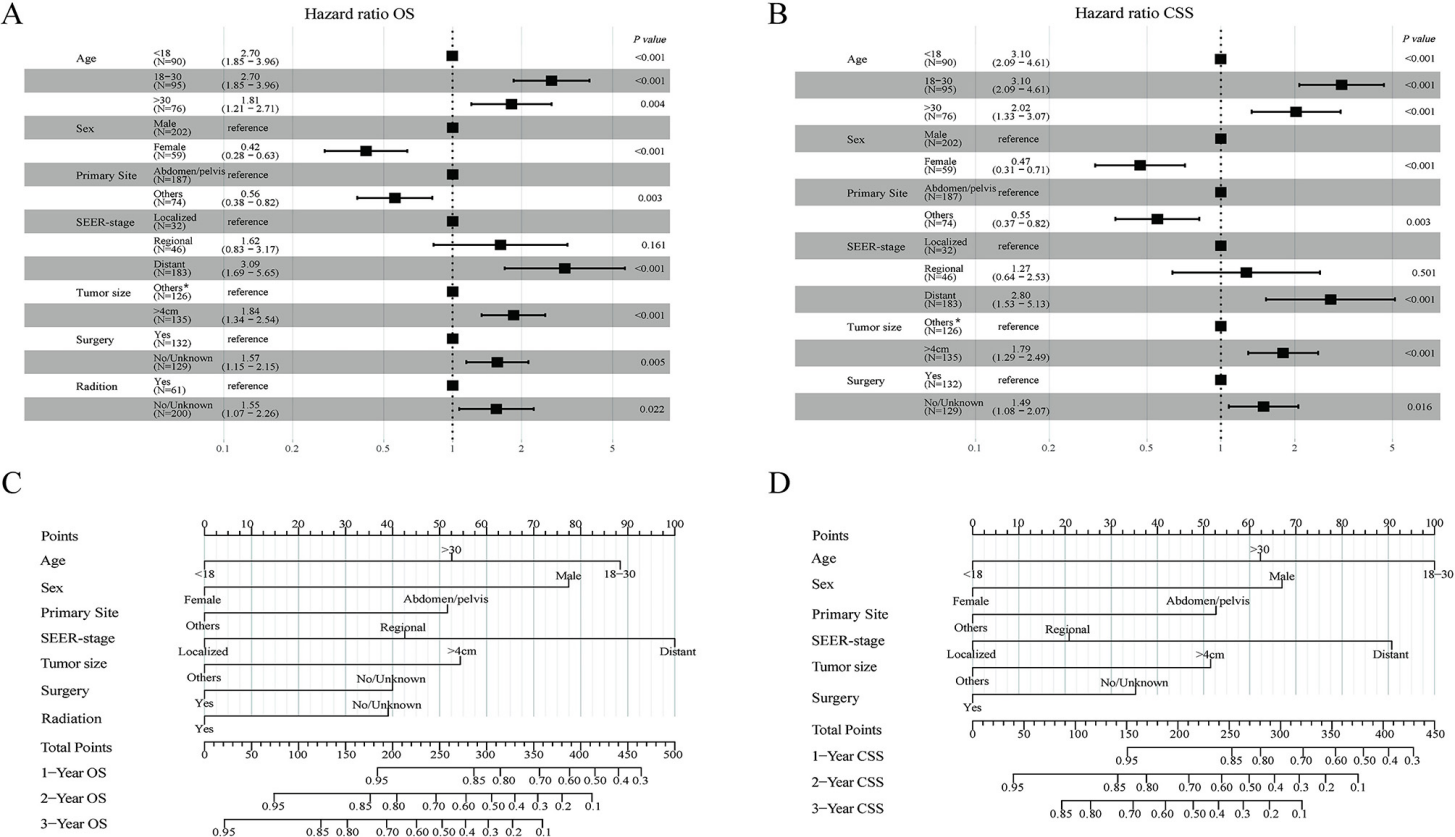
**Forest plot demonstrating the LASSO-Cox regression model for OS (A) and CSS (B) in the training cohort and the nomogram for predicting 1-, 2- and 3-year OS (C) and CSS (D).** *Others include ≤ 4 cm and unknown. OS: Overall survival; CSS: Cancer-specific survival; LASSO: Least absolute shrinkage and selection operator.

Before employing the LASSO-Cox regression, Spearman’s correlation among all screened variables was examined for collinearity. Figure 2A shows the findings of the correlation analysis. To avoid overfitting when selecting significant features, we utilized LASSO-Cox regression to find the best coefficient for every prognostic component on the least partial probability deviation basis and to construct coefficient curves from logarithmic (lambda) series (Figure 2C and 2E) [21]. Six clinical features (age, sex, primary site, tumor size, SEER-stage, surgery), and seven clinical features (age, sex, primary site, SEER-stage, surgery, tumor size, radiation), of the clinical features, were identified as independent predictors in the CSS and OS models, respectively, following the LASSO-Cox regression minimum requirements with 10-way cross-validation (Figure 2B and 2D). Then, we included these potential parameters in forest plots to develop survival prediction models-based nomograms for OS and CSS (Figure 3A and 3B). This investigation demonstrated that age in the OS model and SEER stage in the CSS model is the clinical characteristics most strongly related to prognosis. The scores for the selected factors may be used to determine the survival probability rapidly and rationally for individual patients using the nomogram (Figure 3C and 3D).

### Validation of the nomogram

**Figure 4.**
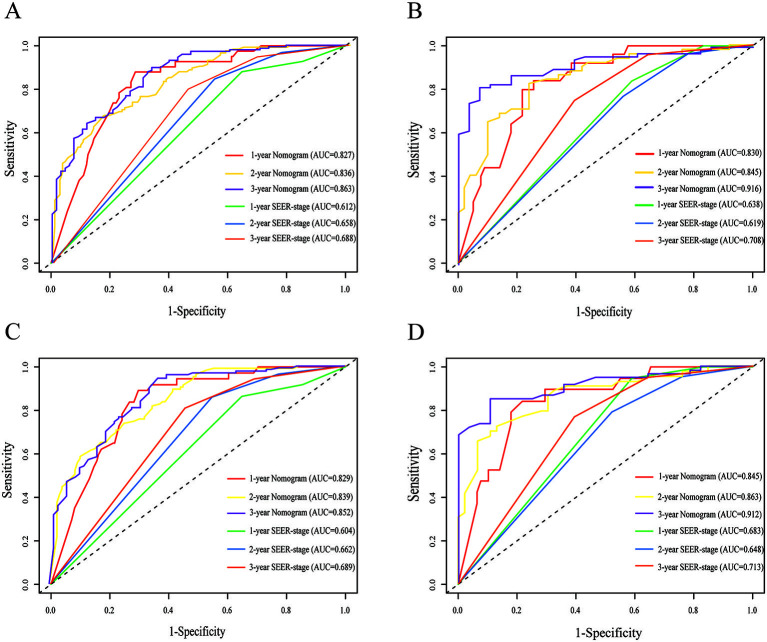
**Time-dependent ROC curves comparing the use of the nomogram and SEER-stage system to predict the 1-, 2- and 3-year OS and CSS in the training cohort (A and C) and the internal validation cohort (B and D).** ROC: Receiver operating characteristic; AUC: Area under the curve; OS: Overall survival; CSS: Cancer-specific survival.

The nomogram predicted OS at 1-, 2-, and 3-years had a C-index (training cohort: 0.770; validation cohort: 0.790) greater than that of the SEER-stage system (training cohort: 0.643; validation cohort: 0.631). Similarly, the 1-, 2-, and 3-year CSS accuracy for model prediction was considerably greater than the SEER-stage system, with C-indices of 0.731 vs 0.631 in the training cohort and 0.723 vs 0.631 in the validation cohort. Compared to the SEER-stage system, the 1-, 2-, and 3-year AUC values of the nomograms were all above 0.8, indicating that the models had much superior predictive ability (Figure 4). The calibration plots revealed concordance between expected survival and actual survival. The 1-, 2-, and 3-year OS (Figure S1) and CSS (Figure S2) predictions for the training and validation cohorts using the nomogram models were accurate. With a broad range of favorable threshold probabilities, nomogram-based CSS or OS model decision curve analysis exhibited good clinical usefulness and predictive accuracy for predicting 1-, 2-, and 3-year survival (Figures S3 and S4).

### A dynamic online survival estimate calculator

Our nomograms for CSS and OS in DSRCT patients are accessible to researchers and clinicians at https://dsrct.shinyapps.io/CSSforDSRCT/ and https://dsrct.shinyapps.io/OSforDSRCT/. By inputting clinical characteristics and reviewing the tables and figures that the web server generates, it is easy to compute the projected survival probability over time.

### Risk stratification based on the nomogram

**Figure 5.**
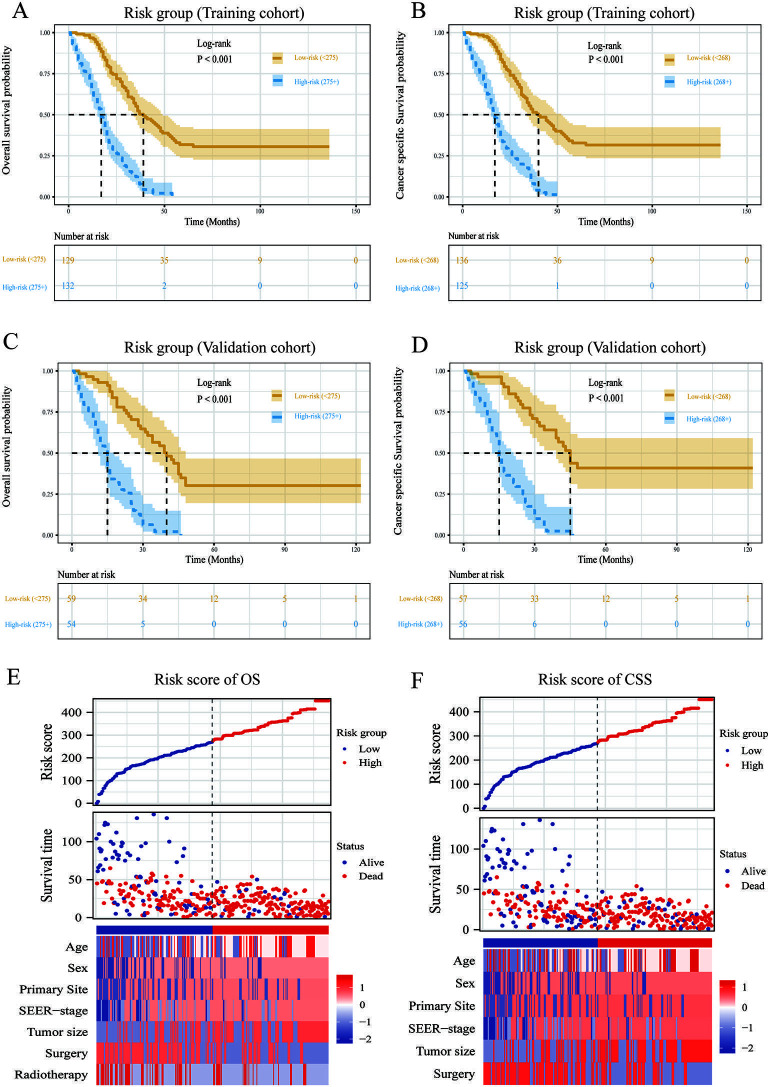
**Overview of risk-stratification system according to risk points calculated by nomogram.** OS and CSS analysis of patients with DSRCT in the training cohort (A and B) and validation cohort (C and D). The distribution of clinicopathological features in different risk groups for OS (E) and CSS (F). OS: Overall survival; CSS: Cancer-specific survival; DSRCT: Desmoplastic small round cell tumor.

According to the optimal cutoff point identified by the Survminer tool, the nomogram-classified patients were separated into low-risk and high-risk groups (OS model: 275 points; CSS model: 268 points). Significantly distinct (*P* < 0.001) survival curves for CSS and OS were seen among patients in different risk categories, lending preliminary support to the nomogram and risk categorization approach (Figure 5A–5D). Additionally, risk factor-related heatmaps were used to display the abnormal clinical features distribution by OS (Figure 5E) and CSS (Figure 5F) risk groups.

**Figure 6.**
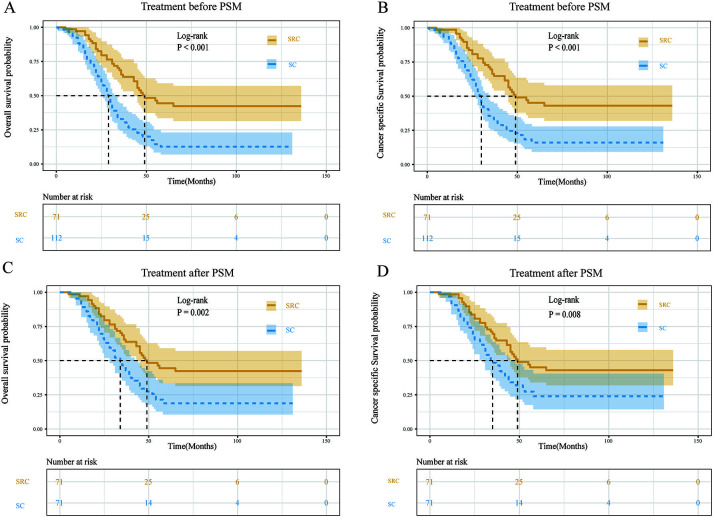
**Comparison of survival differences between treatment strategies analyzed by PSM.** Survival curves of two groups before and after matching analysis in OS (A and C) and CSS (B and D). PSM: Propensity score matching; OS: Overall survival; CSS: Cancer-specific survival; SRC: Triple-therapy (surgery plus radiation and chemotherapy).

### Subgroup analysis of treatment strategies

PSM analysis, which can effectively control for confounding variables, was utilized to investigate further the influence of various therapies on prognosis [22]. Moreover, data analysis before and after PSM is shown in Tables S1. Given the greater weight of surgery in our prediction models and the widespread availability of chemotherapy for DSRCT, this research evaluated the prognostic impact of triple-therapy (surgery plus radiation and chemotherapy) compared to surgery plus chemotherapy. Prior to the matched analysis, we found that triple-therapy had a superior OS and CSS than surgery plus chemotherapy, with a median survival time of 49 vs 29 months and 49 vs 30 months, respectively (Figures 6A and 6B). After matching for patient characteristics, triple-therapy continued to give a substantial advantage in OS and CSS, with median survival times of 49 vs 34 months and 49 vs 35 months, respectively (Figure 4C and 4D).

## Discussion

DSRCT, a rare malignant soft tissue tumor with a poor prognosis, is extremely aggressive. The median survival time is only 2 years, and cases exceeding 40 months are rarely reported [23]. DSRCT is characterized by tiny spherical cell nests and surrounding sclerotic connective tissue that may concurrently exhibit neural, epithelial, and mesenchymal markers; the molecular marker is the EWS-WT1 fusion protein [24, 25]. At present, it is believed that the EWS-WT1 presence might be the essence of DSRCT proliferation, which leads to the activation of downstream biological pathways, such as vascular endothelial growth factor, platelet-derived growth factor, and transforming growth factor beta, which ultimately results in tumor proliferation [26]. Currently, the therapy management of DSRCT focuses mostly on ES; nevertheless, DSRCT is more prevalent in young men (20–30 years; roughly 4:1 male to female ratio), resulting in a greater societal cost [27, 28]. Clinicians need a specialized and reliable survival risk assessment system to predict prognosis and support therapeutic decision-making.

The low incidence of DSRCT certainly creates a critical challenge. The SEER database is one of the dependable sources of information on cancer treatment, diagnosis, survival, and incidence in the United States [29] and has garnered great interest in cancer research and provides exceptional benefits for examining rare malignancies [30–32]. In this work, we collected information on DSRCT patients from SEER (*n* ═ 374) and found 7 and 6 clinical characteristics linked with OS and CSS, respectively. According to the training and validation sets, these characteristics are reliable predictors of survival in the DSRCT. To our knowledge, this is the first extensive study that uses the SEER to create a nomogram prediction model for OS and CSS of DSRCT. The clinical value of the prediction models was further improved with the help of a live, web-based survival risk calculator.

According to the research, age is a significant factor in determining the prognosis of DSRCT, and the median age of onset is approximately 25 years [1–3, 14]. This research revealed that patients between the ages of 18 and 30 had the worst prognosis, followed by those above the age of 30, and those under the age of 18 had the best prognosis. In addition, the age component was the top and second weighted fraction of CSS and OS, respectively, in our risk prediction model. The previous study has shown that more males than females suffer from malignancy [1–3]. The male-to-female patient ratio was approximately 3 to 1, and male patients had poorer prognosis. Recent studies have shown that androgen receptor can promote the progression of DSRCT, and high levels of circulating dihydrotestosterone are associated with poor prognosis [33]. Therefore, androgen receptor-based treatment may improve the survival rate of male patients, which is worthy of further prospective study. Consistent with earlier research, the present analysis revealed that the abdomen and pelvis were the most prevalent disease locations linked to a poorer prognosis [3, 10]. The size of the tumor is a significant determinant in the prognosis of many solid tumors [34–36], as it reflects the severity of the tumor load to some degree. Consistent with clinical data, our research indicated that patients with a significant tumor burden (tumor size >4 cm) had shorter OS and shorter CSS. In the present DSRCT research area, the absence of a widely recognized, accurate prognostic staging system is a significant issue. The combined summary stage is a unique characteristic of the SEER database, unlike the TNM staging method. It is the most fundamental method of classifying how far cancer has progressed from its origin and is separated into three stages: local, regional, and distant. It is intended to assist practical application [37]. In this research, the combined summary stage (SEER-stage) was used to investigate the fundamental biological features of DSRCT, and the findings indicated that most patients were already in the distant stage at the time of their first diagnosis.

The current research examines the relationship between medical treatments and patient outcomes in more depth, revealing that surgery is essential for increasing both OS and CSS. For individuals with DSRCT, aggressive surgical therapy and sensitive chemotherapy regimens are the cornerstone of treatment. Cytoreductive surgery and R0 resection have been demonstrated to enhance patient outcomes in prior research [11, 38]. Chemotherapy is important in all DSRCT patients who can tolerate chemotherapy, and this research indicates its extensive use in real world. Unfortunately, the ES chemotherapy regimen did not significantly enhance DSRCT patients’ survival rates [7, 14]. In addition, hyperthermic intraperitoneal chemotherapy is an optional local treatment for patients with residual disease after surgery, which may further improve their prognosis [39, 40]. Whole abdominal radiotherapy is mainly used for postoperative CS or palliative treatment to further improve the local control rate and relieve symptoms, but gastrointestinal reactions are more apparent, and there are certain controversies in its clinical application [18, 41]. The current study showed a significant improvement in OS in patients who received radiation therapy but not in CSS. However, the latest retrospective study by Subbiah et al. showed no survival benefit from adjuvant radiotherapy [42]. In view of the time-varying differences in the clinical application of radiotherapy, the above conclusions need to be clarified by further prospective studies. Given the uncertainty and limitations of current treatment options, our study uses the PSM to investigate whether triple-therapy can further prolong survival. The results showed that after balancing the confounding factors between the two groups, patients who received triple-therapy still had better OS and CSS than those who received surgery plus chemotherapy. Therefore, more aggressive triple-therapy can be tried for patients with good general status, but special attention must be paid to the associated toxic side effects.

This study employed LASSO-Cox regression to provide a comprehensive framework for assessing survival risk. Furthermore, the model’s accuracy was much higher than the SEER-stage system employing a multidimensional validation. The limitations of the present investigation must be acknowledged. Due to the retrospective nature of this study, missing patients from the SEER database were not included, which might have led to a sampling error. In addition, the SEER registry missed essential clinical factors, such as performance score, chemotherapy details (regimens and cycles), and second-line treatment, which may have diminished the model’s predictive accuracy over the long run. Third, there may be some collinearity among the variables included in the model, which may lead to overfitting in the model. Finally, the SEER database lacks information on progression-free survival and relapse survival, which may limit the model’s applicability.

## Conclusion

In summary, through a detailed analysis of patient records in the SEER database between 2000 and 2019, we found that multiple clinical factors have an independent impact on the OS and CSS in patients with DSRCT. In addition, we improved clinical progress by creating and validating highly accurate prediction models. Potentially better outcomes might be achieved with triple-therapy.

## Supplemental Data

Supplementary data are available at the following link: https://www.bjbms.org/ojs/index.php/bjbms/article/view/8633/2706.
